# World Health Organization World Hand Hygiene Day, 5 May 2024. SAVE LIVES: clean your hands campaign: promoting knowledge and capacity building on infection prevention and control, including hand hygiene, among health and care workers

**DOI:** 10.1186/s13756-024-01391-8

**Published:** 2024-04-12

**Authors:** Claire Kilpatrick, Ermira Tartari, Miranda Deeves, Didier Pittet, Benedetta Allegranzi

**Affiliations:** 1 Infection Prevention and Control Unit and Hub, Department of Integrated Health Services, World Health Organization (WHO), Geneva, Switzerland; 2 Faculty of Medicine, University of Geneva, Geneva, Switzerland

## Abstract

The World Health Organization’s (WHO) World Hand Hygiene Day continues to *“bring people together and accelerate hand hygiene action at the point of care in health care to contribute to a reduction in health care-associated infections and the achievement of safer, quality health care for all”.*

Many countries are demonstrating strong engagement and advancements in scaling-up infection prevention and control (IPC) strategies and actions, but overall, the progress is slow, and gains are at risk. In 2021, only four out of 106 countries (3.8%) had all minimum requirements for IPC in place at the national level.^1^ In multiple WHO surveys, training and education was the weakest component of IPC programmes around the world both at the national and facility level.^1^ This is reflected in lack of standardized IPC curricula for pre-graduate courses (e.g. medicine, nursing, midwifery), in-service training, and for post-graduate specialization, leading to discontinuous delivery of IPC training and lack of experts and mentors, and of career pathways for IPC professionals.

That is why the World Hand Hygiene Day 2024 focuses on **“promoting knowledge and capacity building of health and care workers through innovative and impactful training and education, on infection prevention and control, including hand hygiene”.** Furthermore, the 2024 SAVE LIVES: Clean Your Hands campaign coincides with the need for countries to rapidly consider implementation of the first ever global strategy on IPC adopted by all countries in 2023,^2^ and supported by a forthcoming global action plan and monitoring framework, which include a key strategic direction related to IPC education and training.

WHO is calling on all IPC practitioners, health and care workers, policy and decision makers, alongside the public, to join activities and celebrations for World Hand Hygiene Day 2024 by sharing knowledge of why hand hygiene is still important - because it helps stop the spread of harmful germs in health care (Fig. 1).

Find all campaign assets here: https://www.who.int/campaigns/world-hand-hygiene-day/world-hand-hygiene-day-2024.

## Figure legend

Fig. 1 5 May 2024: World Health Organization World Hand Hygiene Day campaign slogan and banner.

## Footnotes

1 Global report on infection prevention and control. Geneva, Switzerland: World Health Organization; 2022 (https://www.who.int/publications/i/item/9789240051164).

2 Global strategy on infection prevention and control. Geneva, Switzerland: World Health Organization; 2023 (https://www.who.int/publications/m/item/global-strategy-on-infection-prevention-and-control).

